# Single center experience with wrapping of the dilated ascending aorta

**DOI:** 10.1186/s13019-015-0371-1

**Published:** 2015-11-20

**Authors:** Tomasz Plonek, Andrzej Dumanski, Rafal Nowicki, Wojciech Kustrzycki

**Affiliations:** Department of Cardiac Surgery, Wroclaw Medical University, Borowska 213, 50-556 Wroclaw, Poland

**Keywords:** Aneurysm, Aorta, Wrapping

## Abstract

**Background:**

External wrapping is a surgical technique performed in patients with a dilated ascending aorta. The aim of this study is to present the mid-term results of wrapping of the dilated ascending aorta.

**Methods:**

34 patients (mean age: 64.4 ± 10.8 years, 21 males) with a dilated ascending aorta were operated on at a single cardiac surgery center using a wrapping technique. The aortas were wrapped with 32–36 mm straight Dacron vascular prostheses. The aortic wall was not excised in any of the patients. Wrapping was performed concomitant to other cardiac surgery procedures in 30 patients (88 %), which involved surgery on the aortic valve in 28 patients (82 %).

**Results:**

The mean follow-up time was 19.5 ± 8.3 months (median: 18 months, range: 12–36 months). None of the patients died or had aortic complications during the hospital stay and the follow-up period. A rethoracotomy had to be performed due to excessive postoperative bleeding in two patients. One patient was diagnosed with a transient ischemic attack on the 4th postoperative day, while another had respiratory failure requiring prolonged intubation. No redilatation of the ascending aorta or dislocation of the wrap was noticed in any of the patients.

**Conclusions:**

According to our study, external wrapping of the ascending aorta has good short-term results and may be regarded as a safe surgical option for patients with a moderately dilated ascending aorta.

## Background

Aortic wrapping is an operative technique which can be used for the treatment of a dilated ascending aorta. Robicsek et al. published the results of an external reinforcement of the ascending aorta in 1971 [1]. The technique was also used in vascular surgery to treat abdominal aortic aneurysms and first described in the early 50s [2]. This procedure leads to a reduction of the diameter of the vessel by applying a corset made of an artificial material, i.e. a Dacron vascular prosthesis. So far, there have been few reports describing postoperative results of an isolated wrapping technique [3–8]. Most studies report the results of wrapping with concomitant aortoplasty, where the excessive aortic wall is either resected or plicated, followed by a reinforcement using an external material [9]. Isolated wrapping (without aortoplasty) is a procedure that is mainly utilized in patients with a moderately dilated aorta (a diameter of 40–55 mm) undergoing other cardiac surgery operations.

We present the results of the use of the wrapping technique without concomitant aortoplasty. The aim of this study is to assess the early mortality, morbidity and the change in the diameter of the ascending aorta in patients operated on using this technique.

## Methods

### Patients

The study was approved by the Local Ethics Committee (No. KB 791/2012). 34 patients with a nondissected dilated (>40 mm) ascending aorta were operated on using the wrapping technique. The procedure was performed in patients that did not suffer from aortic wall calcification or dissection. Patients characteristics are presented in Table 1.

**Table 1:** Patients characteristics

Number of patients	34
Males (%)	21 (62 %)
Age	64.4 ± 10.8 years (median: 64.5, range: 29–82)
Preoperative diameter of the ascending aorta	47.2 ± 4.5 mm (median: 46, range: 41–60 mm
Bicuspid aortic valve	6 (17.6 %)
Euroscore 2	5.37 ± 5.11 % (median: 3.24 %, range: 0.96–25.52 %)
Type 2 diabetes	8 (23.5 %)
Hypertension	28 (82 %)

Wrapping was performed as an isolated procedure in 4 (12 %) patients. In the remaining 30 patients (88 %), wrapping was performed as a concomitant procedure to other cardiac surgery operations, which involved aortic valve procedures in 28 patients (82 %) (Table 2).

**Table 2:** Concomitant cardiac procedures performed in patients who underwent the wrapping procedure

Concomitant cardiac surgery procedures	No. of patients
AVR	18 (53 %)
Wrapping alone	4 (12 %)
AVR + CABG	4 (12 %)
AV reapir	3 (9 %)
AV repair + CABG	1 (3 %)
AVR, MVR (reoperation)	1 (3 %)
AVR + CABG+ ASD closure	1 (3 %)
MV repair	1 (3 %)
MV repair + TV repair + CABG	1 (3 %)

*AVR* aortic valve replacement, *CABG* coronary artery bypass grafting, *MVR* mitral valve replacement, *ASD* atrial septal defect, *MV* mitral valve, *TV* tricuspid valve, *AV* aortic valve

The preoperative aortic diameter was measured using both CT-angiography and transthoracic echocardiography. During the follow-up period, the ascending aorta was assessed using transthoracic echocardiography.

### Operative technique

**Fig. 1:**
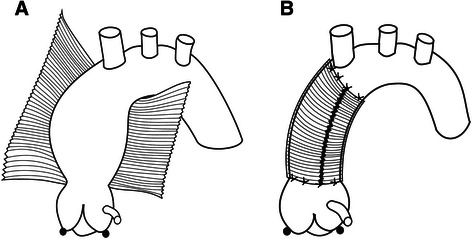
A schematic presentation of wrapping of the dilated ascending aorta. **a** A longitudinally cut straight Dacron vascular prosthesis passed under the aorta. **b** A wrapped aorta

Following the opening of the pericardial sac, the aorta was gently dissected, separated from the pulmonary artery and mobilized from the level of sinotubular junction up to the innominate artery. In all patients wrapping was performed on a beating heart with the patient connected to the extracorporeal circulation. During reperfusion, shortly after releasing the aortic clamp a 32–36 mm straight Dacron vascular prosthesis was cut longitudinally and placed around the dilated segment of the ascending aorta. Subsequently, the edges of the prosthesis were approximated using two continuous 3–0 Prolene sutures running from the proximal and distal ends of the prosthesis and tied in the middle of the wrap. The systolic arterial pressure was kept below 80 mmHg. To prevent the dislocation of the wrap, the proximal and distal ends of the prosthesis were sutured to the surface of the aorta using several 5–0 Prolene stitches (Fig. 1).

## Results

There were no intraoperative complications in any of the patients and there were no problems associated with applying the aortic wrap. The intra and perioperative data are presented in Table 3. None of the patients died during the early postoperative period. One patient was diagnosed with a transient ischemic attack (TIA) on the fourth postoperative day. Another patient had respiratory problems and required prolonged intubation (for 7 days). No permanent neurologic damage was observed in any of the patients. A rethoracotomy was performed in two patients due to excessive postoperative blood loss. Four patients (12 %) were diagnosed with acute kidney injury (AKI). However, they did not require any renal replacement therapy. No wound infections were noted and all patients were discharged home in a good general physical condition.

**Table 3:** Intra and perioperative data

Time of the procedure	3 h 32′ ± 1 h 04′ (median: 3 h 15′, range: 1 h 25′–6 h 25′)
Aortic cross-clamping time	55′ ± 31′ (median: 58′, range: 0′ – 1 h 48′)
Extracorporeal circulation time	1 h 39′ ± 46′ (median:1 h 35′, range: 0′- 3 h 38′)
Inotropic support	6 (17.6 %)
The mean postoperative drainage during the first 24	540 ml ± 400 ml (median:390 ml, range:190 ml – 1750 ml)
Blood transfusion	12 (35 %)

There were no cases of early postoperative aortic complications. The postoperative echocardiography (5–7th postoperative day) revealed no redilatation or dissection either in the wrapped or unwrapped portions of the aorta in any of the patients. The diameter of the wrapped segment of the aorta was on average 30.7 ± 1.5 mm (median:31 mm, range:26–33 mm).

The mean follow-up time was 17.9 ± 10 months (median: 18 months, range: 6–36 months). None of the patients died during the follow-up period. There were no cases of aortic dissection, redilatation or vascular prosthesis dislocation and none of the patients required reoperation. There was no significant difference in the diameter of the aortic root −0,1 ± 0,9 mm (median: 0, range: −2 - 2 mm) and tubular part of the ascending aorta 0,1 ± 0,7 mm (median: 0, range: −1 - 1 mm) measured using echocardiography shortly after the procedure and during the follow-up period.

## Discussion

A dilated tubular part of the ascending aorta is a common finding in patients with aortic valve pathologies. The threshold for the replacement of the ascending aorta is 55 mm in patients without other comorbidities, i.e. the Marfan syndrome or a bicuspid aortic valve [10]. To date, there is no agreement whether a moderately dilated aorta should be replaced or left intact using the watch-and-wait approach. A recent study by Rylski et al. revealed that the aorta dissects at a diameter smaller than the above mentioned threshold [11]. This means that an earlier intervention, especially in patients that undergo other cardiac surgery operations, may be reasonable.

The replacement of the ascending aorta using a supracoronary interposition graft prolongs the aortic cross-clamping and extracorporeal circulation time compared to an isolated aortic valve replacement. Moreover, it increases the risk of bleeding from a suture line compared to standard aortotomy. A less invasive technique may be suitable in patients who have moderately dilated aortas that undergo cardiac surgery procedures. External wrapping does not prolong the cross clamping time and reduces blood loss compared to the replacement of the aorta [3, 12, 13]. The technique itself is easy and does not require long training. It is a convenient and safe procedure that can be used in a selected group of patients whose aortas are not calcified and are not very dilated (>60 mm). In our opinion, aortic wrapping should be performed when the patient is connected to the extracorporeal circulation, as this allows better control of aortic pressure and may save the patient in case of a damage to the aortic wall and subsequent massive bleeding.

Several studies presented good mid-term results of the isolated wrapping of the ascending aorta (without additional aortoplasty) [3–8]. According to the results of a metaanalysis of the wrapping technique the early mortality in patients undergoing this operation was 0,4 % [9]. The two largest studies presenting the results of an isolated wrapping technique reported no early or late aortic related mortality [3, 5]. Moreover, a recently published biomechanical analysis reports that external wrapping decreases the stress and strain in the aortic wall and may also decrease the risk of aortic dissection [14]. The incidence of aortic complications after wrapping procedure is low [9]. However, a few cases of complications have been published. These were usually associated with the dislocation of the aortic wrap or an aortic root redilatation [15–17]. Based on the experience of surgeons who routinely use this technique, the vascular prosthesis used during wrapping should be anchored to the aorta proximally and distally to prevent it from dislocating [5]. Moreover, a moderately dilated aortic root (45–55 mm) cannot be left intact during the wrapping procedure and should be either replaced or wrapped to avoid the risk of the development of an aortic root aneurysm.

External wrapping can be safely performed in patients undergoing other cardiac procedures. We believe that it is a reasonable option for patients who have a moderately dilated aorta. It does not prolong the cardiac ischemic time and can lower the risk of aortic complications in patients whose aortic diameters are too small to qualify for standard aortic replacement procedures.

## Conclusions

The results of this study indicate that external wrapping of the ascending aorta has good short-term results. None of the patients died or suffered from aortic complications during the follow-up. Therefore, aortic wrapping may be regarded as a safe surgical option for patients with a moderately dilated ascending aorta.
